# Experimental chlorine partitioning between forsterite, enstatite and aqueous fluid at upper mantle conditions^☆^

**DOI:** 10.1016/j.gca.2013.05.026

**Published:** 2013-11-15

**Authors:** Alessandro Fabbrizio, Roland Stalder, Kathrin Hametner, Detlef Günther

**Affiliations:** a Institute of Mineralogy and Petrography, University of Innsbruck, Innrain 52f, 6020 Innsbruck, Austria; b ETH Zürich, Lab of Inorganic Chemistry, Wolfgang-Pauli-Str. 10, 8093 Zürich, Switzerland

## Abstract

Cl partition coefficients between forsterite, enstatite and coexisting Cl-bearing aqueous fluids were determined in a series of high pressure and temperature piston cylinder experiments at 2 GPa between 900 and 1300 °C in the system MgO–SiO_2_–H_2_O–NaCl–BaO–C±CaCl_2_±TiO_2_±Al_2_O_3_±F. Diamond aggregates were added to the experimental capsule set-up in order to separate the fluid from the solid residue and enable in situ analysis of the quenched solute by LA-ICP-MS. The chlorine content of forsterite and enstatite was measured by electron microprobe, and the nature of hydrous defects was investigated by infrared spectroscopy. Partition coefficients show similar incompatibility for Cl in forsterite and enstatite, with *D*_Cl_^fo/fl^ = 0.0012 ± 0.0006, *D*_Cl_^en/fl^ = 0.0018 ± 0.0008 and *D*_Cl_^fo/en^ = 1.43 ± 0.71. The values determined for mineral/fluid partitioning are very similar to previously determined values for mineral/melt. Applying the new mineral/fluid partition coefficients to fluids in subduction zones, a contribution between 0.15% and 20% of the total chlorine from the nominally anhydrous minerals is estimated.

Infrared spectra of experimental forsterite show absorption bands at 3525 and 3572 cm^−1^ that are characteristic for hydroxyl point defects associated with trace Ti substitutions, and strongly suggest that the TiO_2_ content of the system can influence the chlorine and OH incorporation via the stabilization of Ti-clinohumite-like point defects. The water contents for coexisting forsterite and enstatite in some runs were determined using unpolarized IR spectra and calculated water partition coefficients ${D_{{\text{H}}_{2}\text{O}}}^{\text{fo}/\text{en}}$ are between 0.01 and 0.5.

## 1 Introduction

The role of halogens in subduction zones is poorly understood, despite the fact that they are significant components of dehydration fluids released during subduction and as recycled elements into the mantle. Eclogitic rocks that have experienced devolatilization with little or no interaction with external fluid sources can be viewed as relevant analogs for crustal material that may be transferred back into the mantle. Recycled eclogite has a minimum of 100–200 ppm Cl (Philippot et al., 1998). For the Izu arc a slab fluid composition containing Cl = 0.94 ± 0.25 wt.%, F = 990 ± 270 ppm and H_2_O = 25 ± 7 wt.% was estimated at arc front depths (Straub and Layne, 2003), suggesting that the slab becomes strongly depleted in Cl and water in subduction zones. In contrast, much of the subducted F appears to be returned to the deep mantle, implying efficient fractionation of Cl and H_2_O from F during subduction process. For back-arc lavas from Lau Basin, Scotia Sea and Mariana the ratio Cl/H_2_O of the slab fluid component was estimated giving equivalent salinity up to 19 wt.% NaCl (Kent et al., 2002). It is estimated that ∼80% of the Cl in the Manus arc-type submarine quenched glasses was added directly from subducted slab-derived fluids (Sun et al., 2007). Serpentinite rocks from the Betic Cordillera (Spain) and the fluid inclusions trapped within them preserve a record of serpentinite dehydration during progressively deeper stages of subduction (Kendrick et al., 2011). Antigorite in serpentinite preserves chlorine signature close to the sedimentary marine pore fluid, assuming that sedimentary marine pore fluids and seawater are responsible for serpentinisation (Kendrick et al., 2011). The first saline fluids released during serpentine breakdown are enriched in Cl (∼40 wt.% NaCl equiv.) and continued serpentine breakdown contributes to the Cl enrichment of back arc basin basalts (Kendrick et al., 2011). These data provide strong evidence that chlorine is a major component (present at weight percent levels) in the slab fluids.

The presence of aqueous fluids in subduction zone environments implies that chlorine can be partitioned between three broad families of phase (minerals, silicate melts, and aqueous fluids). The transfer of chlorine in subduction zones will depend on its relative partitioning between mineral–fluid, mineral–melt, and fluid–melt. At high pressure aqueous fluids and hydrous melts tend to be completely miscible and a distinction between them cannot be made. The critical pressure in the simple system MgO–SiO_2_–H_2_O is at rather high pressure (>10 GPa, Stalder et al., 2001; Melekhova et al., 2007), but in more complex systems (e.g., eclogite–water, Kessel et al., 2005) it ranges around 5 GPa. Addition of salt, however, generally tends to decrease the solubility of silicate material in the fluid and thereby shifts the critical endpoint to higher pressures (Aranovich and Newton, 1996).

Experimentally determined mineral/fluid partition coefficients for chlorine in the system forsterite–enstatite–pyrope–H_2_O–MgCl_2_ at 1100 °C and 2.6 GPa are in the range 10^−3^–10^−5^ (Bernini et al., 2012) and demonstrate that aqueous fluids efficiently sequester chlorine from anhydrous silicate minerals. Consequently, these fluids have a strong ability to extract Cl from the upper mantle. Experimentally determined partition coefficients of halogens between mantle minerals and basaltic melts under upper mantle conditions (Beyer et al., 2012; Dalou et al., 2012) revealed that halogens are always incompatible in mantle minerals, and that compatibilities of F and Cl are generally ordered as *D*^Cpx/melt^ > *D*^Opx/melt^ > *D*^Grt/melt^ > *D*^Ol/melt^ > *D*^Plag/melt^.

In this study we focus on Cl partition coefficients between forsterite, enstatite and coexisting Cl-bearing fluid for conditions relevant to the upper mantle (2 GPa, 900–1300 °C). A combination of electron microprobe and LA-ICP-MS was used to analyze Cl in experimental phases and FTIR to characterize OH bearing defects in forsterite and enstatite. Furthermore, the water contents for coexisting forsterite and enstatite were determined and the influence of Ti and F on Cl and H_2_O partitioning by the stabilization of humite-type defects is investigated. The water contents determination and the investigation of the influence of Ti and F on chlorine and water partitioning represent also the main differences between this study and the work of Bernini et al. (2012). The new experimental chlorine partition coefficients were used to estimate the contribution of chlorine contents coming from mantle minerals in subduction zones.

## 2 Experimental and analytical methods

### 2.1 Starting materials and sample preparation

The starting materials for the experimental runs consisted of a suite of synthetic MgO–SiO_2_–H_2_O–NaCl–BaO–C±TiO_2_±Al_2_O_3_±CaCl_2_±MgF_2_ powders with varying chlorine content from 0.45 to 11 wt.% (Table 1). Each synthetic powder was prepared in three steps. First, an initial composition was produced using powders of Mg(OH)_2_ and SiO_2_. Two aliquots of this material were doped with different amounts of BaTiO_3_ to obtain starting mixtures rich and poor in TiO_2_. Barium was added as an internal standard for quantification of LA-ICP-MS analyses. Barium is a highly incompatible element in olivine and enstatite, and therefore resides preferably in the fluid or melt phase. These starting mixtures were ground in an agate mortar under ethanol for 1 h and then in an automatic milling machine for 1 h. Then, NaCl was added to the grounded initial powders to obtain the desired chlorine concentration. The doped starting mixtures were ground again in the agate mortar under ethanol for 1 h to achieve chemical homogeneity. One final mixture was also doped with Al_2_O_3_, a second one with CaCl_2_ instead of NaCl, and a third one with MgF_2_ (Table 1).

As sample containers we used Pt-capsules with an outer diameter of 3.0 mm. A Pt-ring with an inner diameter of 2.1 mm and a height of 1 mm was inserted into the Pt-capsule. Then 2 μl H_2_O were added to the capsule, corresponding to 5.6–11.0 wt.% of the starting powder + water system. The capsule was then filled with ∼5 mg of synthetic diamond crystals (grain size 20 μm) kindly provided by Servsix GmbH (Karlstein, Germany), and with 20–35 mg of the starting material powder. The capsule was welded while cooled in water, and the weight of the capsule was compared before and after welding to ensure that no water was lost during welding. The sealed capsule was squeezed to a final cylindrical shape with a length of 5.5–6.5 mm and then held at 120 °C for 24 h, in order to check whether the capsule was sealed. Care has to be taken that: (1) the volume of the fluid or the melt exceeds the volume of the pore space in the diamond layer to ensure homogeneous pressure distribution within the capsule; (2) the experimental charge with its diamond layer is placed in the hotspot of the experimental assembly to prevent unwanted crystal growth within the diamond trap (Aerts et al., 2010). The pore space between the diamond crystals was preserved at high pressure-temperature conditions, so that the fluid was able to communicate with the solid starting material throughout the experiment (Stalder et al., 1998). During quenching the dissolved silicate material precipitated into the pore space between the diamond grains and thus was physically separated from the residual phases.

### 2.2 Experiments

Experiments were carried out in an end-loaded piston–cylinder apparatus at 2 GPa and temperatures in the range of 900–1300 °C for durations between 6 h and 1 week at Innsbruck University. From the outside to the inside, concentric cylindrical assemblies consist of a lead foil wrapped around a talc cell, a Pyrex cylinder, a graphite furnace, and inner pieces of crushable alumina sleeves. Run temperature was controlled to within ∼1 °C of the set point using Pt/Pt_90_Rh_10_ thermocouples. The range in temperature across the length of the capsules during the experiments is estimated to be less than 20 °C. No pressure correction was applied to the EMF. To ensure no temperature drift, power output was monitored during the runs. Each experiment was initiated by gently increasing the sample pressure to approximately 0.25 GPa. It was then heated to the initial temperature (*T^i^*) at a rate of 15 °C/min and the pressure was increased during heating until the final pressure was reached. To grow relatively large, high quality crystals necessary for FTIR analyses, synthesis experiments involved different thermal regimes (Table 2). (a) The sample was heated directly to the final temperature (*T^f^*) and held for the duration of the run. (b) The sample was held to *T^i^* for 1 h and then slowly cooled to *T^f^* at a rate between 6 and 50 °C/h and held at this temperature for the requested time. (c) Olivine and enstatite seeds were added to the starting mixture and then run was performed using the cooling strategy (b) described above. (d) The sample was cooled from *T^i^* to *T^f^* and then immediately quenched. (e) The sample was held at *T^i^* for 1 h and then slowly cooled to an intermediate temperature (*T^m^*), held at this temperature for 24–33 h, and then cooled at *T^f^* and held for the requested time. Using method (b) and (e), crystals in excess of 300 μm in length were synthesized. The aim of the different strategies was to understand if the growth rate influences the chlorine incorporation into the crystals. Strategy (d) implies a total experimental time 4–5 times shorter than that of most of the other experiments, thus providing a proof for equilibrium conditions. The runs were terminated by switching off the power and the experiments were quenched rapidly (∼50 °C/s). After gradual decompression, the charge was removed from the assembly. To check for potential leakage the recovered capsule was weighed and pierced. When pierced the pH of the extruding fluid was checked with indicator paper, the checked fluid was in all cases neutral (pH 7), indicating that the possibly dissolved alkalis are balanced by chlorine. Subsequently, the pierced capsule was dried in an oven at 120 °C (upon drying, salt crystals formed at the opening of the capsule) and weighed again. Usually, the weight loss upon drying corresponds to 90–100% of the initial water content. Experimental run times were 6–100 h at or above 1100 °C and 1 week for runs at 900 °C. Some experiments were conducted without a diamond trap in the Pt-capsule in order to leave a larger volume for the run products, which were subsequently used for FTIR measurements.

All recovered capsules were embedded in epoxy and ground by hand. As soon as the trap became visible, the sample was impregnated with epoxy to prevent destruction during preparation. Later, the capsule was ground until its maximum cross section was exposed.

### 2.3 Analysis of run products

#### 2.3.1 Electron microprobe

**Fig. 1:**
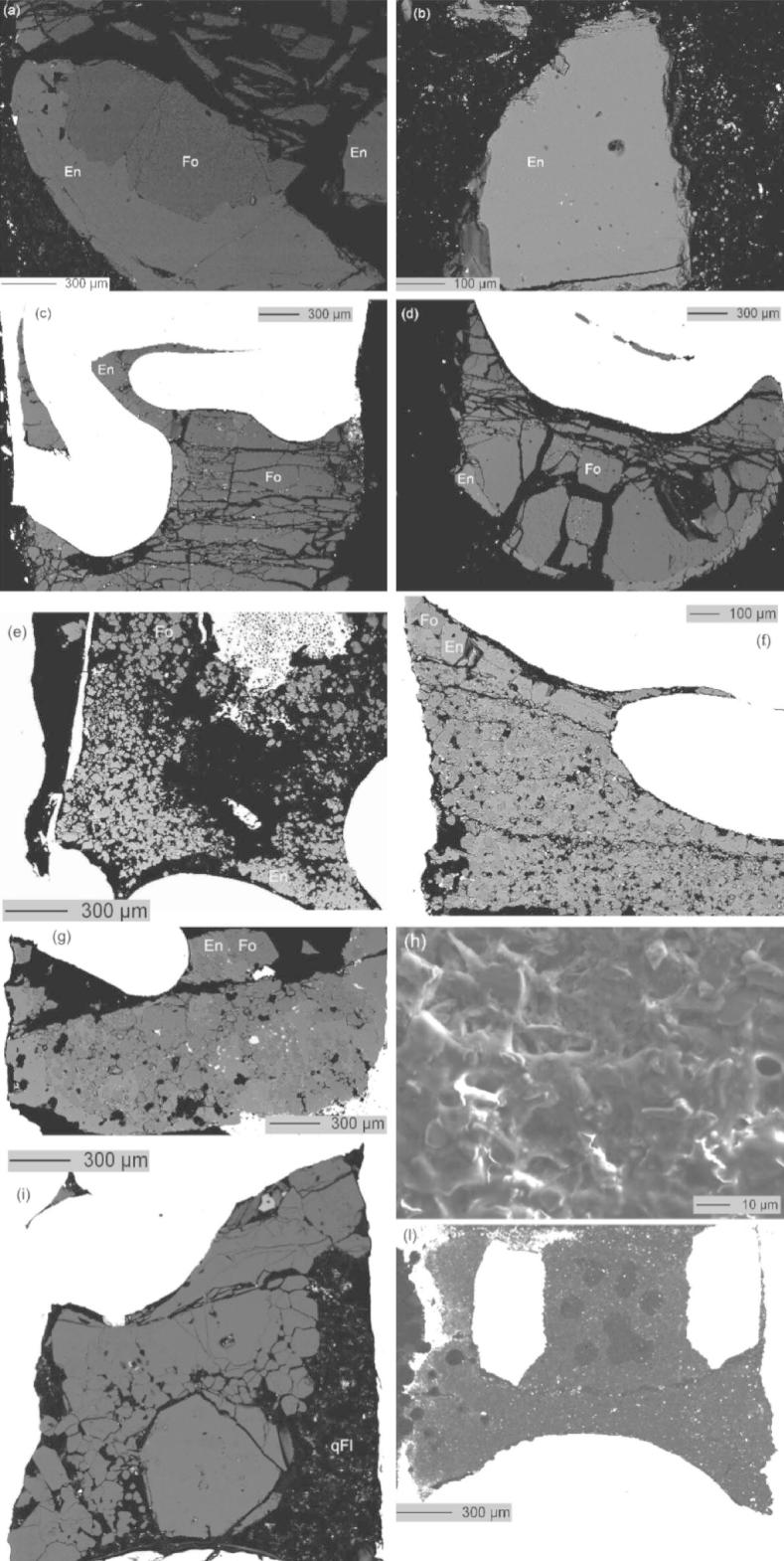
BSE images of the samples and of a diamond trap after the run. Sample Cl-19 (a) and (b); (c) sample Cl-20; (d) sample Cl-21; (e) sample Cl-1; (f) sample Cl-2; (g) sample Cl-3. The BSE images of the samples show the absence of fluid inclusions inside the crystals and the absence of beam damage on the surface of the crystals after the electron microprobe analysis for Cl. The bright zone in (c)–(g) and (i) is the Pt capsule. En = enstatite (light gray); Fo = forsterite (dark gray). (h) Globular materials between diamonds precipitated from the fluid phase during quenching from high *P*–*T* (1200 °C, 2 GPa). (i) Repetition of run Cl-21 showing the quenched fluid (qFl), note that forsterite and enstatite are indistinguishable due to the contrast/luminosity that was adjusted to put in evidence the quenched fluid. (l) BSE image of the diamond trap shown in (h) after laser ablation analysis. The circular holes of 120 μm are laser ablation pits now filled by epoxy. The bright zones inside and outside the diamond trap are the Pt-ring and the Pt-capsule, respectively.

The electron microprobe measurement on forsterite and enstatite were carried out using a Jeol Superprobe 8100 at Innsbruck University. The crystals were analyzed with an electron beam energy of 15 keV, a beam current of 10 nA, a focused beam, and counting times of 20 s (Si, Mg, Ca in enstatite), 120 s (Ca in forsterite). Subsequently, Ba, Ti, Na, Cl, Al, and F were measured with a beam current of 400 nA, and 240 s counting time. BSE images of the samples are reported in Fig. 1. A detailed explanation of the analytical protocol to measure chlorine concentrations is reported in Appendix A. Quartz (Si), diopside (Ca and Mg), barite (Ba), rutile (Ti), aegirine (Na), atacamite (Cl), corundum (Al), and F-topaz (F) were used as standards. Replicate analyses were carried out on the same sample (20–30 points for forsterite and enstatite in each sample) to check for compositional homogeneity. Limits of detection were 2 ppm for Cl, 6 ppm for Na, 18 ppm for Ba and 9 ppm for Ti and 5 ppm for Al. Associated errors on the measured values were less than 10% for Cl, less than 5% for Na, ∼20% for Ti, less than 1% for Al in enstatite, whereas for Al in forsterite and for Ba were more than 100% being always below the limits of detection. Fluorine was not detectable.

#### 2.3.2 LA-ICP-MS

Element concentrations of the fluid trapped in the diamonds were analyzed by LA-ICP-MS at the Laboratory for Inorganic Chemistry – Elemental and Trace Analysis, ETH Zürich, using a 193 nm ArF excimer laser system (Günther et al., 1997) coupled to an ELAN 6100 quadrupole ICP-MS. The NIST SRM610 glass was used to calibrate elements other than Cl. Measured elements were ^23^Na, ^24^Mg, ^25^Mg, ^27^Al, ^28^Si, ^29^Si, ^42^Ca, ^44^Ca, ^47^Ti, ^48^Ti and ^137^Ba. Internal standardization required for element concentration calculations was based on the Ba concentrations (the most incompatible element in this study). A synthetic Cl-bearing basaltic glass was synthesized at 1 atm and 1400 °C and used as a calibration standard for Cl. The composition of the synthetic glass was analyzed by microprobe (Table 3) yielding 0.95 wt.% Cl. More than 600 analyses and also element distribution maps were acquired to verify the homogeneity of the glass. Accuracy was tested by LA-ICP-MS analyses using a NaCl crystal as external standard. The glass was analyzed two times before and two times after performing analyses of the samples of interest and served as external calibration standard. Laser ablation-ICP-MS analysis of Cl has been first reported for fluid inclusion analysis (Heinrich et al., 2003). The achievable accuracy and precision using Cl as internal standard or detecting this element in brine inclusions has been reported in Heinrich et al. (2003). The high ionization potential of 12.8 eV leads to higher limits of detection when compared to other trace elements. The analyses shown in this work provide limits of detection on the order of 15–20 mg/kg using a 120 μm crater diameter and a laser repetition rate of 5 Hz. Internal standardization was applied for determination of the Cl concentration by using Na concentrations. Diamond traps (Fig. 1) were measured with the laser focused to 120 μm diameter at repetition rates of 5 Hz. Eight analyses were carried out on the diamond trap for each sample. In order to check the potential influence of mass interferences ^23^Na^12^C and ^35^Cl, both ^35^Cl and ^37^Cl were measured. Since analytical differences between ^35^Cl and ^37^Cl were always below 3%, the influence of this interference was considered negligible.

#### 2.3.3 FTIR

For preparation the respective experimental charges were cut as whole capsule by diamond wire saw to preserve the texture. Unpolarized spectra on randomly oriented forsterite and enstatite crystals were recorded on doubly polished wafers of 150 μm thickness. Sample thicknesses were measured using a Mitutoyo micrometer and are accurate to ±2 μm. Analyses were limited to crystals free of inclusions and cracks, and 10–15 crystals with different orientations were examined. IR spectra were recorded at room temperature in transmission mode using a Bruker Vertex 70 FTIR spectrometer, which was continuously flushed with dried air to minimize water-vapor background, coupled to a Hyperion 3000 microscope. Each spectrum was acquired by 64 scans in the 550-7500 cm^−1^ range with a spectral resolution of 2 cm^−1^.

## 3 Results

### 3.1 Textures of minerals and diamond traps

Experimental conditions and results are summarized in Tables 1–4. Since the aim of this study was the determination of mineral/fluid partition coefficients it is important to judge whether crystals grew subsolidus (and consequently the term fluid can be used) or supersolidus, i.e., in the presence of a hydrous melt. Although the system forsterite–enstatite–water approaches melting at 1400 °C and 2 GPa (Kushiro and Yoder, 1969), it is important to take into account that the presence of NaCl suppresses melting because it lowers the water activity (Aranovich and Newton, 1996, 1997; Shmulovich and Graham, 1996). The temperature of the solidus in the system Mg_2_SiO_4_ + MgSiO_3_ + H_2_O + KCl at 5 GPa increases ∼100 °C as the Cl/(Cl + H_2_O) molar ratio increases from 0 to 0.1, and ∼200–250 °C for an increase from 0 to 0.2 (Chu et al., 2011). Consequently, since most of the experiments have a Cl/(Cl + H_2_O) molar ratio in the range 0.10–0.26 most of the experiments reported here can be considered being subsolidus. For a few runs (Cl-14 to Cl-17) that have a Cl/(Cl + H_2_O) molar ratio in the range 0.01–0.05, it cannot be excluded that melting occurred in an early stage of the run. More constraints for the potential presence of a melt in the experiments with the lowest Cl/(Cl + H_2_O) molar ratios can be placed comparing the textures of the experimental charges. Textures of representative run products are shown in Fig. 1. No particular textural differences are noted between experiments with low Cl/(Cl + H_2_O) molar ratios (⩽0.05) cooled from 1400 °C or held directly at the final temperature (Fig. 1e and f) and those with high Cl/(Cl + H_2_O) molar ratios (⩾0.1) (Fig. 1a–d, g, i). The quenched phase is subhedral and typically displays acicular habit. Phases interpreted to have crystallized in equilibrium with each other in absence of a melt are euhedral to subhedral and typically have the same grain size.

The small amount of quench phase (Fig. 1i) is interpreted to represent the solute quenched from the subsolidus fluid phase. No abrupt increase in the amount of quenched phase with temperature or with decreasing Cl/(Cl + H_2_O) molar ratios was observed, which is interpreted to indicate the absence of a melt in all performed experiments.

### 3.2 Mineral compositions

Run products were mainly composed of forsterite, enstatite, salt and aqueous fluid. Individual phase assemblages are listed in Table 2. The size of the crystals varied between 50 and 300 μm (Fig. 1). Electron microprobe analyses are listed in Table 3. Multiple analyses (2–3) were performed at the core and the rim of each crystal and up to 20 crystals were analyzed in each run. No chemical zoning patterns were observed. In most of the runs forsterite (Fo_100_) coexists with enstatite. Forsterite shows a maximum Cl content of 133 ± 63 ppm and coexisting enstatite has a Cl content of 119 ± 38 ppm.

### 3.3 Fluid compositions

The composition of the aqueous fluid coexisting with forsterite and enstatite was determined with LA-ICP-MS (Table 3). The aqueous fluids of each run are homogenous with respect to their major element composition and contain large amount of silicate components (mainly Si, Mg, Na and Cl). Measured fluid compositions are generally consistent from run to run with the Cl/(Cl + H_2_O) molar ratio of the fluid. Calculated chlorine partition coefficients (mineral–fluid) are listed in Table 3.

**Fig. 2:**
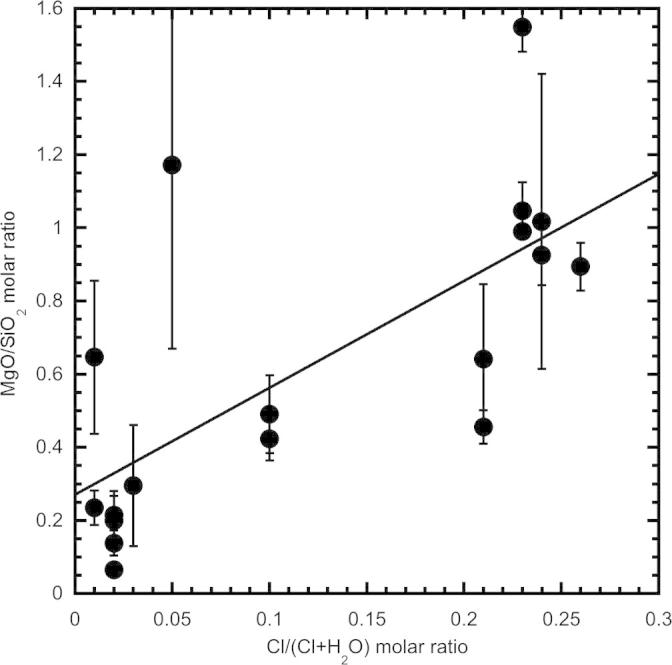
MgO/SiO_2_ molar ratio of the fluids calculated from the LA-ICP-MS analyses (Table 3) vs. the salinity of the fluids expressed as the Cl/(Cl + H_2_O) molar ratio of the starting materials.

**Fig. 3:**
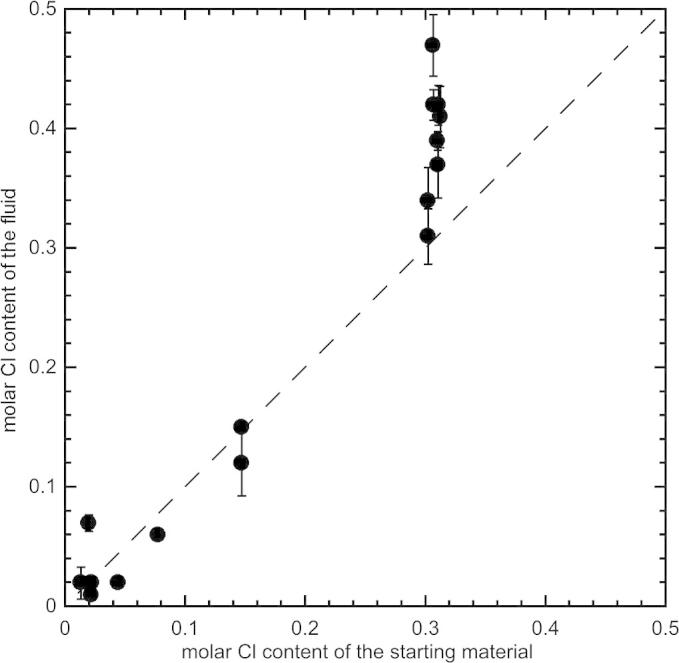
Cl concentration (molar) of the fluids from the LA-ICP-MS analyses (Table 3) vs. the Cl concentration (molar) of the starting materials.

The plot of the MgO/SiO_2_ molar ratios of the fluids vs. the Cl/(Cl + H_2_O) molar ratios of the starting materials (Fig. 2) shows a positive correlation, probably reflecting a decreased solubility of silica at high salinities. The plot of molar Cl contents of the fluids vs. the molar Cl contents of the starting materials (Fig. 3) shows a trend close to the line 1:1, indicating that in most of the experiments the measured Cl contents represent reliable values and that a negligible fraction of Cl was lost during piercing of the capsules.

### 3.4 Minerals–aqueous fluid partitioning

All the reported partition coefficients are wt.%-based. Analyses of the residual crystalline phases (forsterite and enstatite) and the coexisting fluid and the calculated partition coefficients for chlorine (*D*_Cl_^mineral/fluid^ and *D*_Cl_^forsterite/enstatite^) are reported in Table 3. Mass balance calculations suggest minor amounts (∼0.2 wt.%) of rutile in most experiments that was not detected during electron microprobe sessions. In most of the runs the TiO_2_ content of the fluid is around 2000 ppm (Table 3). This result is in agreement with the experimental rutile solubility of 1735 ppm determined for a NaCl brine (10 wt.%) at 1000 °C and 0.5 GPa (Rapp et al., 2010). Run duration and temperature program had no effect on the results.

The quench pH of the fluid was always neutral implying that the compositions of the starting materials have no effect on the pH of the quenched fluid. The neutral pH furthermore implies that equal amounts of alkalis and chlorine were hosted in the fluid after quenching, which is strengthened by the observation of salt precipitation at the capsule opening during drying. Beside water and NaCl, basically all of the material originally dissolved in the fluid was precipitated between the diamond aggregates. The assumption is further strengthened by the observation that the weight loss of H_2_O was always close to 100% at the end of the experiment, and the clear appearance of the water released from the capsule upon piercing.

**Fig. 4:**
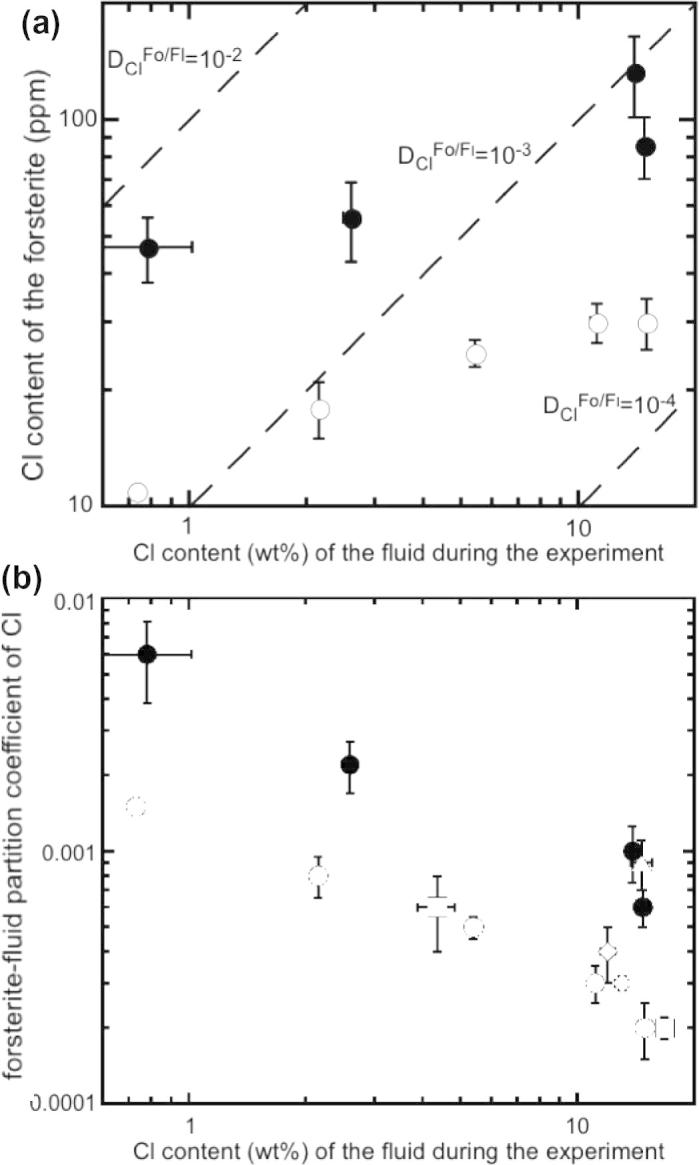
(a) Chlorine content of forsterite (ppm) vs. chlorine content (wt.%) of the fluid during the experiment at 1200 °C, dashed lines represent constant partition coefficients; (b) chlorine partition coefficients obtained at different temperature vs. chlorine content (wt.%) of the fluid during the experiment. Squares: 1300 °C; circles: 1200 °C; diamond: 1100 °C; triangle: 900 °C. Closed symbols represent runs doped with Ca, Al or F. Error bars are ±1*σ*.

The results for forsterite/fluid partitioning at 1200 °C (Fig. 4a) show a strong fractionation of chlorine into the fluid phase. Forsterite crystallized in systems containing Al, Ca or F seems to be able to dissolve up to 80–130 ppm Cl, whereas the maximum chlorine content in forsterite in the undoped system is 25–30 ppm. Forsterite–fluid partition coefficients for Cl at 1200 °C as a function of salt concentration are shown in Fig. 4b. Partition coefficients from experiments at low salinity (i.e., Cl in fluid <5 wt.%) are ∼10^−3^, and at higher salinities (⩾5 wt.%) *D*_Cl_^forsterite/fluid^ is close to ∼10^−4^ and ∼10^−3^ for systems with Al and F. The monotonous decreases of *D*_Cl_^forsterite/fluid^ with increasing salinity could indicate that experiments with more than 5 wt.% chlorine in the fluid were outside the region of application of Henry’s law. Therefore only partition coefficients derived from experiments with less than 5 wt.% chlorine in the fluid are considered for further interpretation. The lack of a significant change in *D*_Cl_^forsterite/fluid^ with salinity higher than 5 wt.% for run times varying from 6 to 39 h at 1200 °C is interpreted to reflect equilibrium among minerals and the aqueous fluid. No significant effect of temperature is recognized in the calculated *D*_Cl_^forsterite/fluid^ from experiments at different temperatures. However, at 1100 °C *D*_Cl_^forsterite/fluid^ increases for Ti-bearing forsterite by a factor of ∼1.3 with respect to Ti-free forsterite, and at 900 °C *D*_Cl_^forsterite/fluid^ increases by a factor of ∼4.5 with respect to the value calculated for the run at 1300 °C. The possible reason is discussed in Section 4.1.

**Fig. 5:**
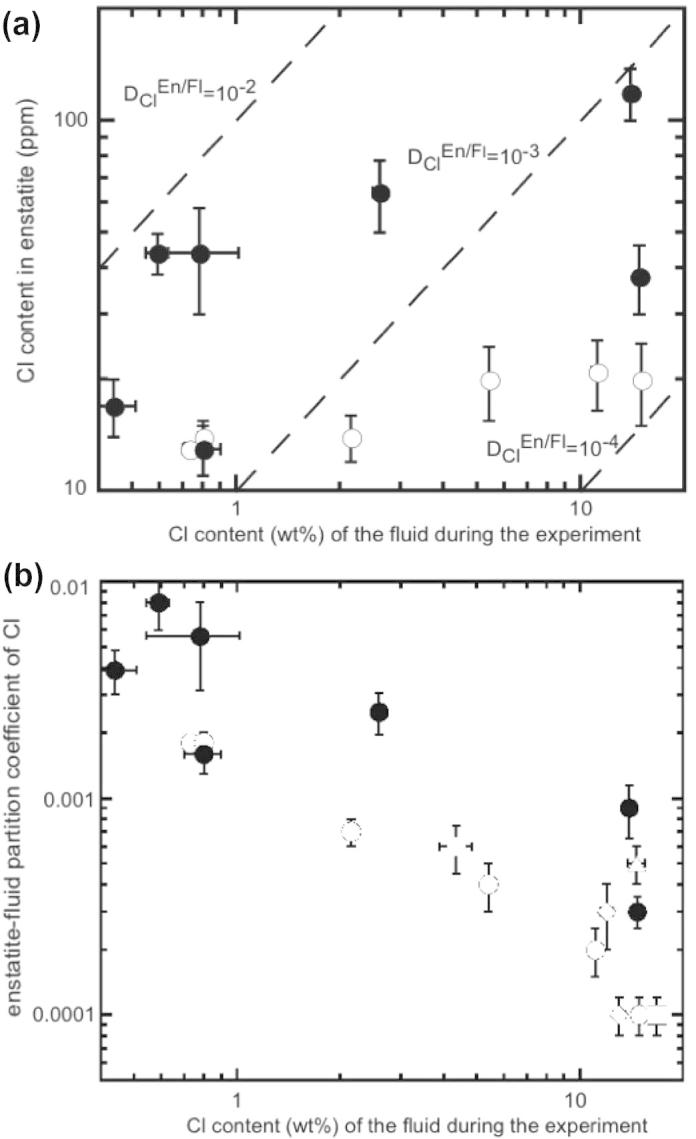
(a) Chlorine content of enstatite (ppm) vs. chlorine content of the fluid (wt.%) during the experiment at 1200 °C, dashed lines represent constant partition coefficients; (b) chlorine partition coefficients obtained at different temperature vs. chlorine content of the fluid (wt.%) during the experiment. Squares: 1300 °C; circles: 1200 °C; diamond: 1100 °C; triangle: 900 °C. Closed symbols represent runs doped with Ca, Al or F. Error bars are ±1*σ*.

The chlorine content of enstatite at a temperature of 1200 °C is shown in Fig. 5a as a function of the chlorine content of the fluid during the experiment. In the simplest chemical system enstatite can dissolve ∼20 ppm Cl, whereas when aluminum, calcium or fluorine are present the solubility of chlorine can increase up to ∼100 ppm. Chlorine partition coefficients for enstatite (Fig. 5b) are close to ∼10^−3^ if the chlorine content of the fluid is less than 5 wt.%. With salinity of the fluid higher than 5 wt.% *D*_Cl_^enstatite/fluid^ tend to decrease to ∼10^−4^. No recognizable dependence on temperature is observed. Enstatites synthesized at lower temperature (900 °C) show *D*_Cl_^enstatite/fluid^ values (5 × 10^−4^) comparable to those synthesized in presence of aluminum or fluorine. In general, both for forsterite and enstatite the *D*_Cl_^mineral/fluid^ values calculated in systems with Ca, Al or F are a factor 3–5 higher compared to values derived from the system free in Ca, Al and F.

**Fig. 6:**
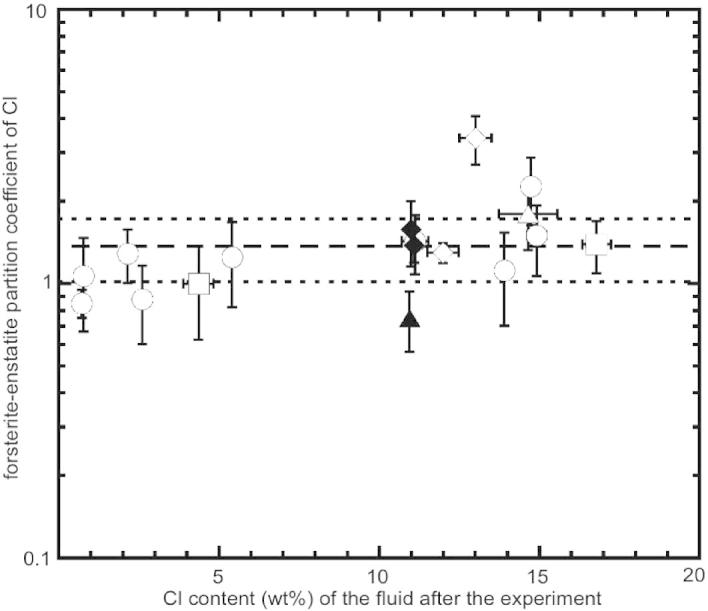
Comparison of forsterite–enstatite partitioning coefficients for Cl from experiments with and without diamond trap as a function of chlorine content of the fluid during the experiment (wt.%). The experiments without diamond trap are plotted as a function of the nominal chlorine content of the starting material. Open symbol: experiment with diamond trap; closed symbol: experiment without diamond trap. Squares: 1300 °C; circles: 1200 °C; diamond: 1100 °C; triangle: 900 °C. Error bars are ±1*σ*. Dashed line represents the mean value of *D*_Cl_^forsterite/enstatite^ calculated from all data and dotted lines represent 1*σ* deviation.

Fig. 6 shows a comparison of forsterite–enstatite partition coefficients from experiments with and without the diamond trap as a function of chlorine content of the fluid after the experiment. Irrespective of the run temperature, in these experiments the Cl concentration in the fluid has no significant effect on the Cl partitioning between forsterite and enstatite. The absence of the diamond trap does not either influence the corresponding *D*_Cl_^forsterite/enstatite^ values. The average *D*_Cl_^forsterite/enstatite^ is 1.43 ± 0.71 implying no significant preference for chlorine in forsterite compared to enstatite.

**Fig. 7:**
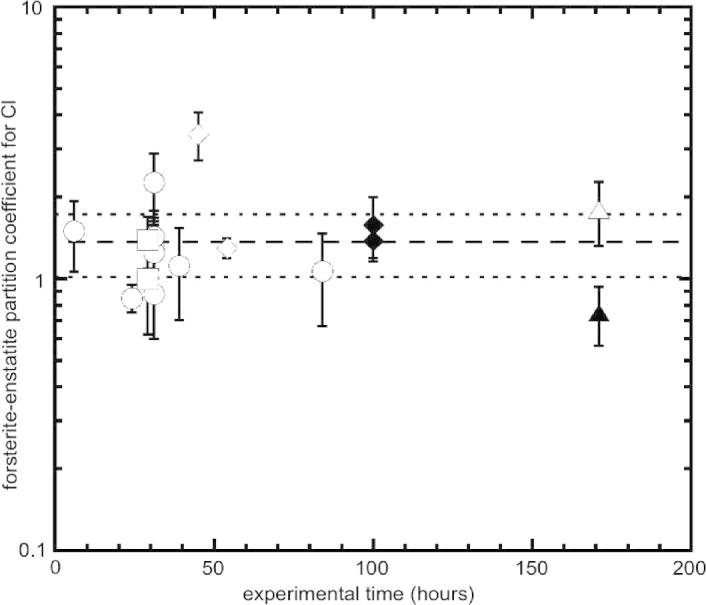
Comparison of forsterite–enstatite partitioning coefficients for Cl from experiments with and without diamond trap as a function of the experimental time. Open symbol: experiment with diamond trap; closed symbol: experiment without diamond trap. Squares: 1300 °C; circles: 1200 °C; diamond: 1100 °C; triangle: 900 °C. Error bars are ±1*σ*. Dashed line represents the mean value of *D*_Cl_^forsterite/enstatite^ calculated from all data and dotted lines represent 1*σ* deviation.

The partition coefficients between forsterite and enstatite are also plotted in Fig. 7 as a function of the experimental time. The longest (171 h) and shortest (6 h) runs show *D*_Cl_^forsterite/enstatite^ values similar to those of most of the experiments performed with the duration of 24–36 h. These observations suggest that chemical equilibrium was attained even at shortest run times.

### 3.5 FTIR spectra for forsterite and enstatite

**Fig. 8:**
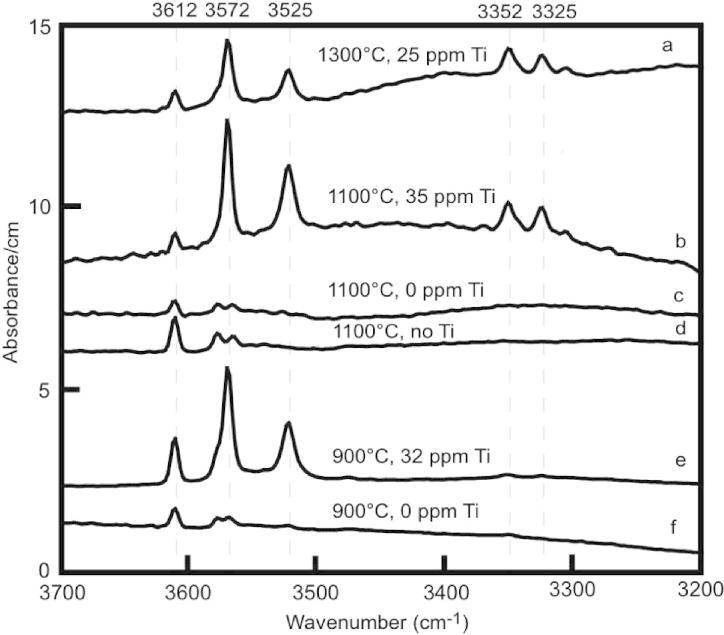
Unpolarized IR spectra of forsterite averaged over 10–15 randomly oriented crystals for each run (a = run Cl-5, b = Cl-7, c = Cl-19, d = Cl-20, e = Cl-21, f = Cl-9). Experimental temperatures and amount of Ti (ppm) in forsterite are reported above each spectra. Spectra are normalized to 1 cm thickness. Vertical dashed lines indicate the positions of OH bands. B.d.l. = below detection limit. No Ti means Ti-free experiment.

**Fig. 9:**
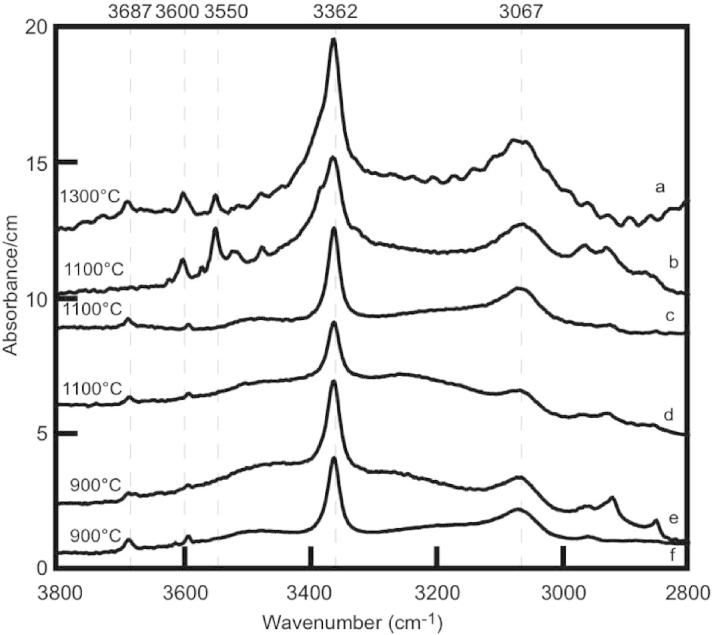
Unpolarized IR spectra of enstatite averaged over 10–15 randomly oriented crystals for each run (a = run Cl-5, b = Cl-7, c = Cl-19, d = Cl-20, e = Cl-21, f = Cl-9). Experimental temperatures are reported above each spectra. Spectra are normalized to 1 cm thickness. Vertical dashed lines indicate the positions of the most prominent OH bands. Absorption features below 3000 cm^−1^ are caused by residues of resin.

Unpolarized IR spectra collected on forsterite and enstatite from selected runs are shown in Figs. 8 and 9, respectively.

Ti-bearing forsterite crystals (Table 3 and Fig. 8) exhibit intense absorption bands at 3525 and 3572 cm^−1^. Minor absorption bands are observed at 3612 cm^−1^ and at 3325 and 3352 cm^−1^. Ti-free forsterite displays also the absorption band at 3612 cm^−1^, but the band at 3572 cm^−1^ tends to disappear and is substituted by two weak peaks at ∼3568 and ∼3580 cm^−1^. The thickness and the average orientation of the samples are comparable and hence the intensity of absorption is related to the amount of OH. The observed spectra indicate that Ti-bearing forsterite incorporates significantly higher amounts of OH compared to Ti-free forsterite. Furthermore, Ti-forsterite from the runs at 900 and 1100 °C exhibits stronger OH-bands than the Ti-forsterite derived from the run at 1300 °C.

In enstatite two strong absorption bands were observed at 3067 and 3362 cm^−1^ (Fig. 9), and minor bands are observed in the range 3475–3690 cm^−1^. Apparently, Ti does not influence the incorporation of OH in enstatite, and the bands between 3475 and 3600 cm^−1^ are probably related to the presence of Al as contaminants. This hypothesis is discussed in Section 4.2.

Since 10–15 unpolarized spectra in each run were collected both for enstatite and forsterite the water contents were quantified applying the method of Kovács et al. (2008). The amounts of water dissolved as point defects in coexisting forsterite and enstatite (Table 4) were calculated using the calibrations of Libowitzky and Rossman (1997) and Bell et al. (1995).

## 4 Discussion

### 4.1 Hydrous defect species in forsterite

Experiments conducted with high amounts of TiO_2_ (Cl-5, Cl-7, Cl-21) show enhanced OH-contents relative to those performed with minor amounts (Cl-9, Cl-19) or without (Cl-20) TiO_2_. For example, samples with the Ti-rich composition have stronger absorption bands at 3525 and 3572 cm^−1^ than other samples at comparable temperature (Fig. 8). This observation is in agreement with the suggestion that absorption bands at 3525 and 3572 cm^−1^ are characteristic for hydroxyl point defects associated with trace Ti substitutions and have been used as fingerprint for Ti-clinohumite-like point defects (Berry et al., 2005). The negative temperature dependence of these absorption bands is also in accord with Hermann et al. (2007). These observations strongly suggest that the TiO_2_ content of the system can influence the chlorine incorporation in forsterite via the stabilization of Ti-clinohumite-like point defects. Subsequently, experiments are in progress to investigate the presence and stabilization of clinohumite lamellae in forsterite in the MgO–SiO_2_–TiO_2_–H_2_O–F–Cl system. The hydroxyl region of the infrared absorption spectra of all samples (Fig. 8) exhibit a band at 3612 cm^−1^ that is attributed to hydrated Si vacancies (Berry et al., 2005; Berry et al., 2007). Several lines of evidence indicate that hydroxyl absorption in olivine with vibrational energies between 3300 and 3400 cm^−1^ is attributable to protons incorporated in association with trivalent cations (Grant et al., 2007a). The development of OH absorptions at 3325 and 3352 cm^−1^ (only observed in forsterite from two experiments, spectra a and b in Fig. 8) may be related to the presence of Cr impurity (Berry et al., 2007), which can be a contaminant in the BaTiO_3_ of the starting material.

Water solubilities in forsterite are related to the presence of the trace element Ti (Table 4 and Fig. 8). Forsterite crystals with a Ti content in the range of 25–35 ppm contain between 18 and 35 wt ppm H_2_O, whereas Ti-free forsterite have less than 10 wt ppm.

### 4.2 Hydrous defect species in enstatite

The IR spectra for enstatite crystals synthesized in run Cl-5 and Cl-7 show minor absorption bands at 3475, 3515, 3550 and 3600 cm^−1^ that Stalder (2004) and Grant et al. (2006) relate to the presence of Al. Aluminum could be an accidental contaminant and the source of this contamination could be from Al_2_O_3_ dust in the vicinity of starting mixture preparation, or even diffusion from the crushable alumina sleeve through the Pt capsule during the high pressure and temperature experiment. However, pure enstatite spectra from other works (Rauch and Keppler, 2002; Mierdel and Keppler, 2004; Grant et al., 2006) exhibit absorption bands at 3475, 3515, 3550 and 3600 cm^−1^ suggesting that other workers may have had similar problems.

Absorption bands at 3067 and 3362 cm^−1^ are assigned to M-site vacancies (Prechtel and Stalder, 2011) and bands at 3592 and 3687 cm^−1^ are associated with Si-vacancies (Prechtel and Stalder, 2011) and their low intensity in the present enstatite crystals is indicative of the rather low synthesis pressure (Prechtel and Stalder, 2011).

Comparison of spectra for enstatite synthesized over the same temperature interval but with different thermal regimes (e.g., Cl-19 vs. Cl-20; Cl-21 vs. Cl-9) show no significant difference in OH defect population confirming that crystal growth rate does not have a significant effect on the concentration of OH defects incorporated into synthetic enstatite (Grant et al., 2006).

The relative uniformity of the spectra for enstatite (with the exception of the OH-peaks due to potential contamination by Al) over the entire range of investigated temperature and composition, strongly suggest that lamellae of monoclinic pyroxene (Mierdel and Keppler, 2004) similar to humite-lamellae in olivine (Hermann et al., 2007) are not stabilized during enstatite growth and crystallization.

The amount of water in enstatite dissolved as OH point defects varies significantly with temperature. Enstatite synthesized at 900 °C contains ∼75 wt ppm H_2_O that is considerably lower than the ∼210 wt ppm for the run carried out at 1300 °C (Table 4). This feature confirms that the high temperature stage at the beginning of the run is not preserved, but annealing as temperatures as low as 900 °C occurs.

### 4.3 Water partitioning

Water partition coefficients between coexisting forsterite and enstatite are between 0.01 and 0.5. The data provide information on the effect of temperature and Ti impurities on *D*_H___2___O_^fo/en^. The increase of water partition coefficients between Ti-bearing olivine and coexisting enstatite from 0.08 at 1300 °C to 0.44 at 900 °C arises from the coupled effect of temperature dependence of OH solubility in enstatite and the enhancing effect of Ti impurities for water incorporation in forsterite. The *D*_H___2___O_^fo/en^ values for Ti-bearing olivine (*D*_H___2___O_^fo/en^ = 0.35 at 1100 °C and *D*_H___2___O_^fo/en^ = 0.44 at 900 °C) are higher than those for Ti-free olivine (*D*_H___2___O_^fo/en^ = 0.08 at 1100 °C and *D*_H___2___O_^fo/en^ ∼ 0.01 at 900 °C), suggesting that the effect of Ti incorporation into forsterite significantly increases the solubility of water in forsterite giving a large increase in *D*_H___2___O_^fo/en^.

Partition coefficients determined in this studycover approximately the same range as previously published values for *D*_H___2___O_^ol/opx^ that vary significantly depending on pressure, temperature, major and trace elements composition. A *D*_H___2___O_^ol/opx^ value = 0.6 was calculated from xenocrysts in a kimberlite (Bell et al., 2004), and a value of *D*_H___2___O_^ol/opx^ = 0.2 under mantle conditions was estimated combining experimental data on the solubility in olivine, with analyses of the water concentrations in a natural orthopyroxene/melt pair(Hirth and Kohlstedt, 1996). Experimental results at 2 GPa and 1380 °C (Koga et al., 2003) and at 1–2 GPa and 1230–1380 °C (Aubaud et al., 2004) give *D*_H___2___O_^ol/opx^ around 0.1, and for a natural lherzolite composition partition coefficients at 2.5 and 4 GPa *D*_H___2___O_^ol/opx^ are 0.15 and 0.2, respectively (Kovács et al., 2012). *D*_H___2___O_^fo/en^ for coexisting forsterite and enstatitesynthesized at 1–2 GPa and 1100–1350 °C in the system MgO–SiO_2_–H_2_O±Al_2_O_3_ (Grant et al., 2006) were between 0.3 and 0.7, and coexisting forsterite and enstatite synthesized at 1.5 GPa and ∼1300 °C in the system albite–forsterite–H_2_O give a mean value of *D*_H___2___O_^fo/en^ ∼ 0.04 (Grant et al., 2007b).

### 4.4 Chlorine–water partitioning in forsterite and enstatite

Partition coefficients of chlorine and water for forsterite (${D_{\text{fo}}}^{\text{Cl}/{\text{H}}_{2}\text{O}}$) and enstatite (${D_{\text{en}}}^{\text{Cl}/{\text{H}}_{2}\text{O}}$) are presented in Table 4. Most of the ${D_{\text{fo}}}^{\text{Cl}/{\text{H}}_{2}\text{O}}$ values are higher than unity whereas ${D_{\text{en}}}^{\text{Cl}/{\text{H}}_{2}\text{O}}$ values are around 10^−1^. Given that coexisting forsterite and enstatite can dissolve comparable amounts of chlorine, the difference between the ${D_{\text{fo}}}^{\text{Cl}/{\text{H}}_{2}\text{O}}$ and ${D_{\text{en}}}^{\text{Cl}/{\text{H}}_{2}\text{O}}$ values reflects the higher water solubility of enstatite with respect to forsterite.

### 4.5 Chlorine partitioning

Partition coefficients of chlorine between the coexisting phases are presented in Table 3. Chlorine partitioning between forsterite and enstatite from the results of 17 experiments gives a mean value of *D*_Cl_^fo/en^ = 1.43 ± 0.71. The average value of *D*_Cl_^fo/fl^ is 0.0012 ± 0.0006 (*n = *14), and for *D*_Cl_^en/fl^ is 0.0018 ± 0.0008 (*n = *18).

If we compare our results with experiments from chlorine partition coefficients between basaltic melt and lherzolite minerals for pressures from 0.8 to 2.5 GPa and temperatures from 1265 to 1430 °C by Dalou et al. (2012), their *D*_Cl_^ol/opx^ of 0.72 ± 0.15 is in close agreement with our results.

Experiments performed in the system forsterite–enstatite–pyrope–H_2_O–MgCl_2_ at 1100 °C and 2.6–3 GPa (Bernini et al., 2008, 2012) give partition coefficients for *D*_Cl_^fo/fl^ between 0.000019 and 0.0016, and *D*_Cl_^en/fl^ between 0.00002 and 0.0012 and a mean value of *D*_Cl_^fo/en^ = 1.40 ± 0.84 in agreement with our value for experiments at 1100 °C of *D*_Cl_^fo/fl^ between 0.0004 and 0.0003, *D*_Cl_^en/fl^ between 0.0003 and 0.0001 and a mean value of *D*_Cl_^fo/en^ = 2.12 ± 0.86 (*n *= 4).

In the synthetic system MgO–SiO_2_–H_2_O–NaCl, *D*_Cl_^en/startmix^ at 2.5 GPa and 1150 °C is estimated to be 0.0026 (Stalder et al., 2008). The fluid composition was not calculated and an estimate for Cl partitioning can be performed considering the bulk starting composition.

Hydrous melting experiments conducted at upper mantle conditions (1–4 GPa; 1000–1380 °C) by Hauri et al. (2006) have given chlorine contents in olivine and orthopyroxene always below the detection limit and so partitioning data involving both phases cannot be obtained.

### 4.6 Chlorine in mantle minerals

Published data on chlorine incorporation in mantle minerals in model systems (Stalder et al., 2008; Bernini et al., 2012; Dalou et al., 2012) and in natural compositions (Scambelluri et al., 2004; Beyer et al., 2012) reveal maximal Cl contents in enstatite of 30 ppm (Stalder et al., 2008) and 24 ppm (Bernini et al., 2008) even in highly saline fluids. The experimental results of Dalou et al. (2012) suggest a positively relationship between Cl in orthopyroxene and its jadeite component, with the Cl content varying from 4 to 148 ppm. These data compare well with the Cl concentrations in the present study, i.e., 20 ppm in enstatite coexisting with forsterite and a highly saline fluid and 119 ppm for enstatite doped with aluminum. These values are similar to the Cl-content of an orthopyroxene from a high-pressure metamorphic (>700 °C, 1.8 GPa) harzburgite converted from an antigorite serpentinite during subduction metamorphism (Scambelluri et al., 2004), where 25 ppm Cl were detected. About 3.1 ± 0.2 (*n *= 4) ppm Cl were measured in synthetic enstatite (Bernini et al., 2012). With the available data no correlation was found between Al_2_O_3_ abundance in pyroxenes and their Cl contents.

Olivine synthesized by Dalou et al. (2012) contains less than 20 ppm Cl, and about 30 ppm were measured in synthetic forsterite by Bernini et al. (2008). These data are in close agreement with our determination of ∼30 ppm Cl in forsterite at salt saturation. On the other hand, lower chlorine concentrations of 3.1 ± 0.9 (*n *= 3) ppm were detected in synthetic forsterite (Bernini et al., 2012). The lower chlorine concentrations found in forsterite (Bernini et al., 2012) can be due to the lack of a trace element (such as Ti) capable of enhance water and consequently chlorine incorporation. Natural olivine from high-pressure serpentinite and from metamorphic harzburgite contain Cl concentrations of 24–120 ppm and 7–18 ppm, respectively (Scambelluri et al., 2004). Chlorine concentration in natural olivine from spinel peridotites and oceanic basalts are lower and fairly homogeneous at 5.7 ± 0.5 ppm (Beyer et al., 2012).

### 4.7 Petrological application

The bulk partition coefficient peridotite/fluid can be calculated assuming that clinopyroxene has a higher *D*_Cl_ than orthopyroxene, with *D*_cpx_/*D*_opx_ of around ∼3.8 based on the data of Hauri et al. (2006) and Dalou et al. (2012), and that spinel and garnet contain only negligible amounts of Cl. In contrast, the bulk partition coefficient peridotite/melt can be calculated based on the data of Dalou et al. (2012). Assuming an average spinel lherzolite composition of 62% olivine, 24% orthopyroxene, 12% clinopyroxene, and 2% spinel (McDonough, 1990), a bulk *D*_Cl_^peridotite/fluid^ = 0.0020 ± 0.0009 and *D*_Cl_^peridotite/melt^ = 0.0040 ± 0.0005 were calculated. Combining the *D*_Cl_^peridotite/fluid^ and the *D*_Cl_^peridotite/melt^ values a bulk partition coefficient fluid/melt of 2.0 ± 0.9 is obtained.

From geochemical considerations (Cl/K, CO_2_/Cl and CO_2_/Nb ratios of melt inclusions from mid-ocean ridge basalts and the estimated K and Nb contents of the upper mantle), the abundance of chlorine in the mantle is estimated to approximately 1 ppm (Saal et al., 2002). Assuming 1 ppm Cl for the depleted MORB mantle, 10% melting of mantle peridotite will produce a basaltic melt with a Cl concentration of about 10 ppm. A fluid liberated from the subducting slab infiltrates the overlying mantle wedge and promotes melting. Assuming that the final hydrous melt consists of 10 wt.% of the aqueous component, the Cl concentration in the fluid and in the melt is 18.2 and 9.1 ppm, respectively. This estimation of Cl concentration in the fluid is favorably comparable to an estimate of 25 ppm obtained using the results of Bernini et al. (2012). It has to be noted that this value is nearly independent on the fluid content of the system, because *D*_Cl_^peridotite/fluid^ is very similar to *D*_Cl_^fluid/melt^ = 2.0 ± 0.9 (this study) and *D*_Cl_^fluid/melt^ = 3.0 ± 0.6 calculated from the data of Bernini et al. (2012). The only relevant parameters are the concentration in the source region, the degree of melting and the fact that Cl is strongly incompatible in nominally anhydrous mantle minerals.

In contrast to this estimation, chlorine concentrations of olivine hosted melt inclusions of Loihi seamount, Hawaii, are much higher and range from 160 ppm to 1.11 wt.% providing evidence for the existence of very Cl-rich brines (possibly saturated in NaCl) at shallow levels in the Loihi magma chamber (Hauri, 2002). The chlorine concentration in back-arc lava glasses from the Lau Basin is in the range 0.008–0.175 wt.% (Kent et al., 2002), 0.03–0.12 wt.% for glass inclusions in olivine from samples across the Kamchatka arc (Churikova et al., 2007), and 0.11–0.84 wt.% for submarine volcanic glasses from the eastern Manus basin (Sun et al., 2007). The Cl concentration for the Izu slab fluid is 0.94 ± 0.25 wt.% (Straub and Layne, 2003) and for the Mariana slab fluid is 1.19 ± 0.09 wt.% (Stolper and Newman, 1994). Consequently, our data show that chlorine hosted in olivine and pyroxene is high enough to explain Cl contents in MORBs. On the other hand, for arc lavas the Cl contribution from the nominally anhydrous mantle source region ranges between 20% and 0.15% of the total Cl content implying that chlorine must be mainly derived from the slab.

## Figures and Tables

**Fig. A1:**
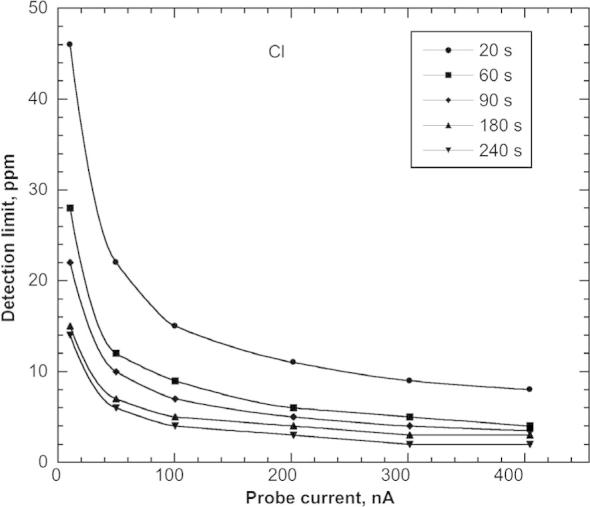
Detection limit for Cl as a function of faraday cup current in nA and peak counting time in seconds. Detection limits were determined for forsterite and enstatite with Jeol software using background statistics and 3 sigma criterion. Typical measurement conditions used in literature for “routine analysis” are faraday cup current less than 50 nA and counting time up to 60 s. Analysis with a faraday cup current of 100 nA and counting time higher than 90 s are “precision analysis”. The analytical conditions used in this study, 400 nA and counting time of 240 s, are for “high precision analysis”.

**Fig. A2:**
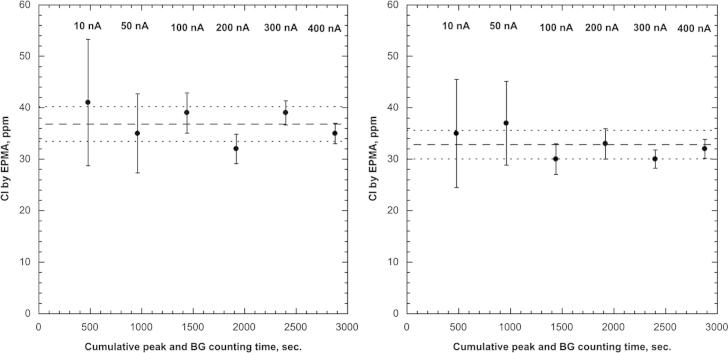
Variation for chlorine concentration in an enstatite crystal (left) and in a forsterite crystal (right) at a single analysis point as a function of the cumulative analysis time (in seconds, sec) and increasing faraday cup current from 10 to 400 nA. With increasing faraday cup current the relative error of the measured concentration is reduced from 30% at 10 nA to 5% at 400 nA. The dashed line show the average concentration for the six consecutive analysis and the dotted lines show the related standard deviation (±2*σ*).

**Table A1:** Comparison between chlorine content in synthetic mantle minerals determined by SIMS (Dalou et al., 2012) and electron microprobe (this study).

Sample	Phase	Cl (ppm) SIMS	Cl (ppm) EPMA
F2p#2a	Opx	26 (2)	22 (4)
F2p#2a	Ol	20 (1)	25 (2)
C8p#2b	Opx	43 (2)	48 (4)
C04#3a	Opx	39 (1)	45 (3)
CC4#7b	Cpx	77 (4)	90 (6)
CF01#16kb1	Opx	5 (1)	5 (4)
CF01#16kb1	Cpx	2 (0.2)	bdl

**Fig. A3:**
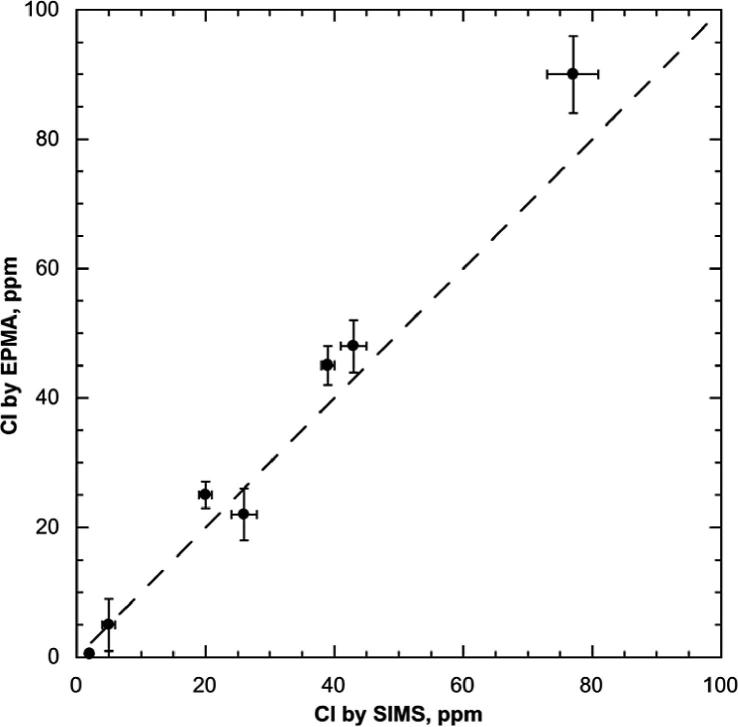
Comparison between chlorine concentrations in synthetic mantle minerals measured by SIMS (Dalou et al., 2012) and EPMA (this study). Error bars correspond to ±2 standard errors for both SIMS and EPMA.

**Table 1:** Composition of starting mixtures normalized to 100 wt.%.

	SiO_2_	TiO_2_	Al_2_O_3_	MgO	CaO	Na_2_O	BaO	Cl	H_2_O	Cl/(Cl + H_2_O)^a^
Cl-1	36.50	0.30	–	35.22	–	1.36	0.58	1.55	24.49	0.03
Cl-2	35.65	0.30	–	34.41	–	2.42	0.57	2.73	23.92	0.05
Cl-3	33.88	0.28	–	32.70	–	4.66	0.54	5.20	22.74	0.10
Cl-4	29.96	0.25	–	28.91	–	9.61	0.48	10.70	20.10	0.21
Cl-5	30.39	0.25	–	29.33	–	9.75	0.48	10.85	18.94	0.23
Cl-6	33.88	0.28	–	32.70	–	4.66	0.54	5.20	22.74	0.10
Cl-7	29.96	0.25	–	28.91	–	9.61	0.48	10.70	20.10	0.21
Cl-8	37.06	0.31	–	35.76	–	0.67	0.59	0.75	24.87	0.02
Cl-9	31.09	0.01	–	30.00	–	9.93	0.02	11.06	17.88	0.24
Cl-10	38.75	0.02	–	37.39	0.34	–	0.03	0.45	23.03	0.01
Cl-11	31.38	0.26	0.58	30.28	–	9.87	0.49	10.98	16.17	0.26
Cl-12	30.91	0.01	–	29.83	–	9.87	0.02	10.99	18.37	0.23
Cl-13	30.82	0.26	–	29.74	–	9.89	0.49	11.00	17.80	0.24
Cl-14^b^	32.25	0.27	–	31.12	0.57	0.60	0.51	0.68	34.00	0.01
Cl-15^b^	37.05	0.31	–	35.76	–	0.68	0.59	0.75	24.86	0.02
Cl-16^b^	37.15	0.31	–	35.85	–	0.68	0.59	0.76	24.66	0.02
Cl-17^b^	37.82	0.01	–	36.50	–	0.69	0.03	0.77	24.18	0.02
Cl-18^b^	30.41	0.25	–	29.35	–	9.75	0.48	10.86	18.90	0.23
Cl-19	31.18	0.01	–	30.09	–	9.96	0.02	11.09	17.64	0.24
Cl-20	30.93	–	–	29.85	–	9.87	–	10.98	18.38	0.23
Cl-21	30.64	0.25	–	29.57	–	9.83	0.49	10.94	18.28	0.23

a Molar ratio.

b Doped also with ∼1000 ppm F.

**Table 2:** Experimental conditions and phases relations of the run products.

Run^a^	*P* (GPa)	*T^i^* (°C)	*T^m^* (°C)	*T^f^* (°C)	Cool rate (°C/h)	Total run duration (h)	Run products identified
Cl-1	2.0	1200	–	1200	–	24	Fo/En/salt/fl
Cl-2	2.0	1400	–	1200	48	31	Fo/En/salt/fl
Cl-3	2.0	1400	–	1200	48	31	Fo/En/salt/fl
Cl-4^b^	2.0	1275	–	1200	48	31	Fo/En/salt/fl
Cl-5	2.0	1400	–	1300	48	29	Fo/En/salt/fl
Cl-6	2.0	1400	–	1300	50	29	Fo/En/salt/fl
Cl-7	2.0	1400	–	1100	50	54	Fo/En/salt/fl
Cl-8	2.0	1400	–	1200	50	31	En/salt/fl
Cl-9	2.0	1400	–	900	50	171	Fo/En/salt/fl
Cl-10	2.0	1400	–	1200	6	84	Fo/En/salt/fl
Cl-11	2.0	1400	–	1200	25	39	Fo/En/salt/fl
Cl-12	2.0	1400	–	1100	50	45	Fo/En/salt/fl
Cl-13	2.0	1400	–	1200	50	6	Fo/En/salt/fl
Cl-14	2.0	1400	–	1200	50	31	Fo/En/salt/fl
Cl-15	2.0	1400	–	1200	25	35	En/salt/fl
Cl-16	2.0	1400	–	1200	50	31	En/salt/fl
Cl-17	2.0	1400	–	1200	50	43	En/salt/fl
Cl-18	2.0	1400	–	1200	50	31	Fo/En/salt/fl
Cl-19^c^	2.0	1400	1200	1100	6/50	100	Fo/En/salt/fl
Cl-20^c^	2.0	1400	–	1100	6	100	Fo/En/salt/fl
Cl-21^c^	2.0	1400	1150	900	6/50	171	Fo/En/salt/fl

Fo, forsterite; En, enstatite; fl, fluid. *T^i^*, initial temperature. *T^m^*, intermediate temperature. *T^f^*, final temperature.

a Run number is coincident with the name of the starting material reported in Table 1.

b Added forsterite and enstatite seeds.

c Experiment performed without diamond trap.

**Table 3:** Analyses of the run products, synthetic glass and minerals by EMPA, fluid by LA-ICP-MS.

Run	Synthetic glass	Cl-1			Cl-2			Cl-3			Cl-4			Cl-5		
		Fl	Fo	En	Fl	Fo	En	Fl	Fo	En	Fl	Fo	En	Fl	Fo	En
SiO_2_	48.14 (75)	4.22 (82)	42.77 (62)	60.07 (44)	1.35 (53)	42.32 (31)	59.74 (59)	3.86 (42)	42.17 (35)	59.96 (20)	7.34 (16)	41.59 (27)	59.66 (33)	8.11 (20)	42.12 (38)	59.53 (9)
TiO_2_	–	0.17 (8)	0.057 (3)	0.44 (3)	0.05 (3)	0.015 (2)	0.12 (1)	0.13 (1)	0.010 (2)	0.24 (1)	0.17 (4)	0.019 (7)	0.13 (3)	0.12 (5)	0.0042 (1)	0.096 (1)
Al_2_O_3_	15.61 (17)	–	–	–	–	–	–	–	–	–	–	–	–	–	–	–
MgO	10.45 (12)	0.84 (44)	58.04 (79)	40.77 (60)	1.06 (81)	58.69 (14)	41.10 (68)	1.10 (10)	58.72 (35)	40.78 (38)	2.24 (22)	57.57 (27)	40.58 (42)	5.70 (40)	58.49 (43)	40.75 (25)
CaO	23.52 (26)	–	–	–	–	–	–	–	–	–	–	–	–	–	–	–
Na_2_O	0.97 (5)		0.021 (3)	0.025 (3)		0.015 (1)	0.025 (9)		0.014 (2)	0.03 (1)		0.023 (4)	0.05 (1)		0.021 (9)	0.045 (5)
Na^a^		0.37 (5)			1.20 (9)			3.70 (17)			8.05 (22)			9.16 (50)		
Cl	0.95 (3)	0.73 (1)	0.0011 (1)	0.0013 (3)	2.15 (2)	0.0018 (6)	0.0014 (4)	5.41 (1)	0.0025 (4)	0.0020 (9)	11.12 (82)	0.0030 (7)	0.0021 (9)	16.79 (91)	0.0032 (6)	0.0023 (9)
Na = Cl^b^		0.47			1.39			3.51			7.21			10.88		
NaCl^c^		1.2			3.54			5.09			18.33			27.67		
Na_2_O^d^		–			–			0.26			1.16			–		
Dissolved silicate^e^		5.23			2.46			5.35			10.87			13.93		
H_2_O^f^	–	93.57	–	–	94	–	–	89.56	–	–	70.80	–	–	58.40	–	–
Total	99.7 (7)	100.0	100.9 (4)	101.3 (4)	100.0	101.0 (3)	101.0 (6)	100.0	100.9 (5)	101.0 (4)	100.0	99.2 (5)	100.4 (3)	100.0	100.6 (3)	100.4 (3)
*D*_Cl_^fo/fl^		0.0015 ± 0.0000			0.0008 ± 0.0003			0.0005 ± 0.0001			0.0003 ± 0.0001			0.0002 ± 0.0000		
*D*_Cl_^en/fl^		0.0018 ± 0.0001			0.0007 ± 0.0002			0.0004 ± 0.0002			0.0002 ± 0.0001			0.0001 ± 0.0000		
*D*_Cl_^fo/en^		0.85 ± 0.20			1.29 ± 0.57			1.25 ± 0.86			1.43 ± 0.70			1.39 ± 0.60		

	Cl-6			Cl-7			Cl-8		Cl-9			Cl-10			Cl-11		
	Fl	Fo	En	Fl	Fo	En	Fl	En	Fl	Fo	En	Fl	Fo	En	Fl	Fo	En
SiO_2_	9.65 (95)	42.71 (21)	60.11 (15)	5.33 (95)	42.55 (57)	59.68 (19)	9.39 (93)	59.54 (32)	2.51 (73)	41.92 (10)	59.23 (44)	4.99 (97)	41.92 (26)	59.54 (59)	20.35 (99)	42.73 (49)	59.25 (51)
TiO_2_	0.21 (3)	0.004 (10)	0.14 (1)	0.16 (5)	0.006 (2)	0.16 (7)	0.19 (2)	0.042 (1)	0.01 (1)	0.00	0.009 (7)	0.03 (2)	0.00	0.006 (2)	0.45 (11)	0.002 (1)	0.27 (7)
Al_2_O_3_	–	–	–	–	–	–	–	–	–	–	–	–	–	–	1.84 (44)	0.00	0.45 (2)
MgO	3.18 (61)	58.74 (23)	41.23 (32)	2.29 (61)	58.60 (54)	40.85 (17)	0.41 (4)	41.21 (21)	1.71 (46)	57.34 (18)	40.19 (36)	2.16 (56)	58.70 (32)	40.75 (33)	12.20 (66)	57.36 (36)	39.64 (42)
CaO	–	–	–	–	–	–	–	–	–	–	–	3.13 (92)	0.021 (7)	0.10 (3)	–	–	–
Na_2_O		0.017 (4)	0.033 (7)		0.11 (3)	0.16 (4)		0.013 (2)		0.013 (7)	0.027 (5)		0.016 (2)	0.013 (1)		–	–
Na^a^	1.73 (35)			6.15 (44)			0.44 (10)		9.00 (46)			0.55 (2)			21.64 (35)		
Cl	4.36 (96)	0.0026 (16)	0.0026 (11)	11.99 (99)	0.0047 (8)	0.0036 (4)	0.80 (5)	0.0014 (3)	14.65 (91)	0.0013 (6)	0.0008 (4)	0.78 (47)	0.0047 (18)	0.0044 (28)	13.91 (27)	0.0133 (63)	0.0119 (38)
Na = Cl^b^	2.82			7.77			0.52		9.49			0.50			9.01		
NaCl^c^	7.18			19.76			1.32		24.14			1.28			22.92		
Na_2_O^d^	–			–			–		–			0.05			12.63		
Dissolved silicate^e^	13.04			7.78			9.99		4.23			10.36			47.47		
H_2_O^f^	79.78	–	–	72.46	–	–	88.69	–	71.63	–	–	88.36	–	–	29.61	–	–
Total	100.0	101.5 (3)	101.5 (5)	100.0	101.3 (5)	100.9 (3)	100.0	100.8 (5)	100.0	99.3 (2)	99.5 (7)	100.0	100.7 (5)	100.4 (7)	100.0	100.1 (8)	99.6 (7)
*D*_Cl_^fo/fl^	0.0006 ± 0.0004			0.0004 ± 0.0001			–		0.00009 ± 0.00004			0.0060 ± 0.0043			0.0010 ± 0.0005		
*D*_Cl_^en/fl^	0.0006 ± 0.0003			0.0003 ± 0.0001			0.0018 ± 0.0004		0.00005 ± 0.00002			0.0056 ± 0.0049			0.0009 ± 0.0005		
*D*_Cl_^fo/en^	1.00 ± 0.75			1.30 ± 0.22			–		1.80 ± 0.95			1.07 ± 0.80			1.12 ± 0.83		

	Cl-12			Cl-13			Cl-14			Cl-15		Cl-16		Cl-17	
	Fl	Fo	En	Fl	Fo	En	Fl	Fo	En	Fl	En	Fl	En	Fl	En
SiO_2_	20.53 (38)	42.23 (55)	59.37 (44)	8.47 (32)	42.18 (39)	59.21 (45)	4.47 (13)	42.68 (13)	59.73 (35)	15.63 (62)	60.02 (70)	12.32 (87)	59.33 (65)	4.75 (52)	58.41 (75)
TiO_2_	0.02 (1)	0.00	0.019 (8)	0.21 (17)	0.0064 (29)	0.19 (5)	0.08 (2)	0.0016 (12)	0.058 (2)	0.15 (4)	0.028 (9)	0.20 (5)	0.041 (1)	0.05 (1)	0.021 (7)
MgO	13.63 (13)	58.22 (17)	40.94 (18)	5.25 (42)	58.11 (42)	40.47 (61)	0.70 (14)	58.30 (26)	40.66 (14)	2.25 (68)	40.13 (64)	1.14 (27)	41.06 (39)	0.64 (20)	41.06 (51)
CaO	–	–	–	–	–	–	0.18 (3)	0.009 (2)	0.064 (2)	–	–	–	–	–	–
Na_2_O		0.019 (3)	0.041 (6)		0.022 (6)	0.039 (7)		0.013 (2)	0.018 (5)		0.021 (6)		0.017 (4)		0.014 (2)
Na^a^	9.04 (21)			10.50 (93)			1.80 (6)			1.07 (63)		1.07 (49)		0.62 (13)	
Cl	13.01 (98)	0.0034 (12)	0.001 (4)	14.92 (59)	0.0030 (9)	0.0020 (10)	2.60 (24)	0.0056 (26)	0.0064 (28)	0.59 (9)	0.0047 (11)	0.44 (14)	0.0017 (6)	0.80 (20)	0.0013 (4)
Na = Cl^b^	8.43			9.67			1.68			0.38		0.29		0.52	
NaCl^c^	21.44			24.59			4.28			0.97		0.73		1.14	
Na_2_O^d^	0.41			0.83			0.12			0.93		1.05		0.14	
Dissolved silicate^e^	34.59			14.76			5.55			18.96		14.71		5.58	
H_2_O^f^	43.97	–	–	60.65	–	–	90.17	–	–	80.07	–	84.56	–	93.28	–
Total	100.0	100.5 (7)	100.4 (6)	100.0	100.3 (8)	99.9 (8)	100.0	101.0 (3)	100.5 (5)	100.0	100.2 (8)	100.0	100.4 (9)	100.0 (9)	99.5 (5)
*D*_Cl_^fo/fl^	0.0003 ± 0.0000			0.0002 ± 0.0001			0.0022 ± 0.0010			–		–		–	
*D*_Cl_^en/fl^	0.0001 ± 0.0000			0.0001 ± 0.0000			0.0025 ± 0.0011			0.0080 ± 0.0041		0.0039 ± 0.0018		0.0016 ± 0.0006	
*D*_Cl_^fo/en^	3.40 ± 1.36			1.50 ± 0.87			0.88 ± 0.56			–		–		–	

	Cl-18			Cl-19		Cl-20		Cl-21	
	Fl	Fo	En	Fo	En	Fo	En	Fo	En
SiO_2_	16.19 (60)	41.34 (82)	58.32 (84)	42.72 (16)	59.90 (40)	42.21 (36)	59.64 (34)	42.21 (45)	59.48 (59)
TiO_2_	0.22 (5)	0.046 (6)	0.23 (3)	0.00	0.00	0.00	0.00	0.0054 (18)	0.19 (5)
MgO	16.81 (39)	59.14 (33)	40.88 (23)	58.75 (41)	40.77 (15)	58.63 (35)	41.01 (24)	58.69 (38)	40.96 (32)
Na_2_O		0.023 (6)	0.041 (5)	0.014 (3)	0.028 (6)	0.014 (4)	0.023 (6)	0.023 (2)	0.053 (9)
Na^a^	10.33 (37)								
Cl	14.74 (45)	0.0086 (31)	0.0038 (16)	0.0018 (4)	0.0013 (2)	0.0019 (8)	0.0012 (4)	0.0030 (6)	0.0040 (18)
Na = Cl^b^	9.55								
NaCl^c^	24.29								
Na_2_O^d^	0.78								
Dissolved silicate^e^	34.00								
H_2_O^f^	41.71	–	–	–	–	–	–	–	–
Total	100.0	100.6 (9)	99.5 (7)	101.5 (5)	100.7 (5)	100.9 (6)	100.7 (5)	100.9 (7)	100.7 (8)
*D*_Cl_^fo/fl^	0.0006 ± 0.0002								
*D*_Cl_^en/fl^	0.0003 ± 0.0001								
*D*_Cl_^fo/en^	2.26 ± 1.25			1.38 ± 0.37		1.58 ± 0.85		0.75 ± 0.37	

Fl, fluid; Fo, forsterite; En, enstatite; dashed line means not analyzed. Values in parentheses represent one standard deviation in terms of least units cited. ^a^Na content of the fluid by LA-ICP-MS. ^b^Assuming that Na = Cl (mole). ^c^NaCl: sum of (b) and Cl content. ^d^Excess of Na dissolved in the fluid as Na_2_O. ^e^Dissolved silicates: sum of the oxides dissolved in the fluid. ^g^Water content: (100 − dissolved silicate − NaCl).

**Table 4:** Water concentrations (ppm) for forsterite and enstatite and calculated partition coefficients.

	Forsterite						Enstatite					Forsterite/enstatite	
	H_2_O^a^	H_2_O^b^	H_2_O^c^	Ti^d^	Cl^d^	e${D_{\text{fo}}}^{\text{Cl}/{\text{H}}_{2}\text{O}}$	H_2_O^a^	H_2_O^b^	H_2_O^c^	Cl^d^	e${D_{\text{en}}}^{\text{Cl}/{\text{H}}_{2}\text{O}}$	d*D*_Cl_^fo/en^	e*D*_H_2_o_^fo/en^
Cl-5	19	18	18	25	32	1.78	250	170	210	23	0.11	1.39	0.08
Cl-7	36	34	35	35	47	1.34	127	70	98	36	0.37	1.30	0.35
Cl-9	7	8	7	0	13	1.86	107	48	78	8	0.10	1.80	0.08
Cl-19	1	2	1	0	18	18	114	81	97	13	0.13	1.38	0.01
Cl-20	8	11	9	0	19	2.11	125	87	106	12	0.11	1.58	0.08
Cl-21	31	36	33	32	30	0.91	100	50	75	40	0.52	0.75	0.44

a Water concentrations (ppm) of forsterite and enstatite calculated using the Bell et al. (1995, 2004) calibration.

b Water concentrations (ppm) of forsterite and enstatite calculated using the Libowitzky and Rossman (1997) calibration.

c Average of water estimation.

d Titanium and chlorine concentrations (ppm) and partition coefficients, *D*_Cl_^fo/en^, as reported in Table 3.

e Calculated chlorine–water partition coefficients, ${D_{\text{fo}}}^{\text{Cl}/{\text{H}}_{2}\text{O}}$ and ${D_{\text{en}}}^{\text{Cl}/{\text{H}}_{2}\text{O}}$, and forsterite–enstatite water partition coefficients, *D*(H_2_O)^fo/en^, using the average of water estimation.

## Appendix A Electron probe microanalysis

The Cl content in synthetic forsterite and enstatite was analyzed with a Jeol Superprobe 8100 at Innsbruck University. Each crystal was analyzed at 15 kV accelerating voltage and faraday cup current of 400 nA. Analytical conditions for Cl analysis are as follows: PETH diffraction crystal, focused beam (beam diameter of 1 μm, excitation volume ∼1.4 μm across, calculated according to Potts (1987)), 480 second total peak and background (BG) counting time (240 s on peak, 120 s BG(+), 120 s BG(−)). The detector PHA setting for Cl were *integral mode*, bias equal to 1716 V, baseline equal to 0.7 V, window equal to 9.3 V, and gain equal to 64. Due to high dead times and pulse height depression (Witter and Kuehner, 2004) these analytical conditions are extreme and should not be used to analyze major elements. For our samples we analyze major elements under routine analytical conditions in a separate analytical sessions, before performing the analysis for Cl. BSE images (Section 2.3.1) of the samples before and after the Cl analysis do not reveal beam damages during analysis. The images demonstrate also the absence of fluid inclusion that could potentially contaminate the Cl signal by X-rays secondary fluorescence. The sample current remains stable during the entire period of analysis, and Cl loss during the analysis was minimal, because of the homogeneity of the studied samples and the high thermal stability of nominally anhydrous minerals. A rastered beam to minimize beam heating was not used in this study, as not all portions of the rastered area experience the same dwell time (Witter and Kuehner, 2004). The ideal Cl-peak position on the PETH diffraction crystal used in our electron microprobe is at an L-value of 151.605 mm. The two background positions were chosen at L-values of 156.605 mm and 149.205 mm, where no peak interferences of other elements were observed. Chlorine was calibrated on a natural atacamite crystal (16.62 wt.% Cl) prior to each analytical session. Detection limits as a function of analysis time and faraday cup current are displayed in Fig. A1.

A synthetic enstatite and a synthetic forsterite crystal were chosen to investigate the variation of the measured chlorine concentrations over time at a single analysis spot (Fig. A2). After long continuous exposure time, each analysis has a total counting time (peak + BG) of 480 s, the chlorine concentration does not vary significantly, suggesting that the damage on the crystals is negligible and that Cl X-rays are not strongly absorbed in the minerals matrix.

A series of experimental samples with Cl contents ranging from 2 to 80 ppm that have been determined by SIMS (Dalou et al., 2012), were analyzed by electron microprobe using the analytical conditions described above. This comparison (see Table A1 and Fig. A3) provide similar results confirming the validity of the chlorine measurements by electron microprobe.
